# No Beneficial Effect of General and Specific Anti-Inflammatory Therapies on Aortic Dilatation in Marfan Mice

**DOI:** 10.1371/journal.pone.0107221

**Published:** 2014-09-19

**Authors:** Romy Franken, Stijntje Hibender, Alexander W. den Hartog, Teodora Radonic, Carlie J. M. de Vries, Aeilko H. Zwinderman, Maarten Groenink, Barbara J. M. Mulder, Vivian de Waard

**Affiliations:** 1 Department of Cardiology, Academic Medical Center, Amsterdam, The Netherlands; 2 Interuniversity Cardiology Institute of the Netherlands, Utrecht, The Netherlands; 3 Department of Medical Biochemistry, Academic Medical Center, Amsterdam, The Netherlands; 4 Department of Pathology, VU University Medical Center, Amsterdam, The Netherlands; 5 Department of Clinical Epidemiology and Biostatistics, Academic Medical Center, Amsterdam, The Netherlands; 6 Department of Radiology, Academic Medical Center, Amsterdam, The Netherlands; University of Amsterdam Academic Medical Center, Netherlands

## Abstract

**Aims:**

Patients with Marfan syndrome have an increased risk of life-threatening aortic complications, mostly preceded by aortic dilatation. In the *FBN1* ^C1039G/+^ Marfan mouse model, losartan decreases aortic root dilatation. We recently confirmed this beneficial effect of losartan in adult patients with Marfan syndrome. The straightforward translation of this mouse model to man is reassuring to test novel treatment strategies. A number of studies have shown signs of inflammation in aortic tissue of Marfan patients. This study examined the efficacy of anti-inflammatory therapies in attenuating aortic root dilation in Marfan syndrome and compared effects to the main preventative agent, losartan.

**Methods and Results:**

To inhibit inflammation in *FBN1* ^C1039G/+^ Marfan mice, we treated the mice with losartan (angiotensin II receptor type 1 inhibitor), methylprednisolone (corticosteroid) or abatacept (T-cell-specific inhibitor). Treatment was initiated in adult Marfan mice with already existing aortic root dilatation, and applied for eight weeks. Methylprednisolone- or abatacept-treated mice did not reveal a reduction in aortic root dilatation. In this short time frame, losartan was the only treatment that significantly reduced aorta inflammation, transforming growth factor-beta (TGF-β) signaling and aortic root dilatation rate in these adult Marfan mice. Moreover, the methylprednisolone-treated mice had significantly more aortic alcian blue staining as a marker for aortic damage.

**Conclusion:**

Anti-inflammatory agents do not reduce the aortic dilatation rate in Marfan mice, but possibly increase aortic damage. Currently, the most promising therapeutic drug in Marfan syndrome is losartan, by blocking the angiotensin II receptor type 1 and thereby inhibiting pSmad2 signaling.

## Introduction

Marfan syndrome is a monogenic connective tissue disorder, caused by mutations in the gene encoding fibrillin-1 (*FBN1*) [1]. The major feature of Marfan syndrome is development of aortic aneurysms, especially of the aortic root, which subsequently may lead to aortic dissection and sudden death [2]–[6].

In a well-known Marfan mouse model with a cysteine substitution in *FBN1* (C1039G), losartan effectively inhibits aortic root dilatation by blocking the angiotensin II type 1 receptor (AT1R), and thereby the downstream production of transforming growth factor (TGF)-β [7]. The destructive role for TGF-β was confirmed since neutralizing TGF-β antibodies inhibited aortic root dilatation in Marfan mice and inhibited the activation of TGF-β-downstream transcription factor Smad2 [7]. Increased Smad2 activation is usually observed in human Marfan aortic tissue and considered crucial in the pathology of aortic degeneration [8]. Even though the response to losartan was highly variable, we recently confirmed the overall beneficial effect of losartan on aortic dilatation in a cohort of 233 human adult Marfan patients [9]. The direct translation of this therapeutic approach from the Marfan mouse model to the clinic, exemplifies the extraordinary power of this mouse model to test novel treatment strategies, which are still necessary to achieve optimal personalized care.

In aortic tissue of Marfan patients, inflammation is observed, which may contribute to aortic aneurysm formation and is the focus of the current study. In the *FBN1* hypomorphic mgR Marfan mouse model, macrophages infiltrate the medial smooth muscle cell layer followed by fragmentation of the elastic lamina and adventitial inflammation [10]. Furthermore, fibrillin-1 and elastin fragments seem to induce macrophage chemotaxis through the elastin binding protein signaling pathway in mice and human Marfan aortic tissue [11], [12]. Increased numbers of CD3^+^ T-cells and CD68^+^ macrophages were observed in aortic aneurysm specimens of Marfan patients, and even higher numbers of these cell types were shown in aortic dissection samples of Marfan patients [13]. In line with these data, we demonstrated increased cell counts of CD4^+^ T-helper cells and macrophages in the aortic media of Marfan patients and increased numbers of cytotoxic CD8^+^ T-cells in the adventitia, when compared to aortic root tissues of non-Marfan patients [14]. In addition, we showed that increased expression of class II major histocompatibility complex (MHC-II) genes, *HLA-DRB1* and *HLA-DRB5*, correlated to aortic root dilatation in Marfan patients [14]. Moreover, we found that patients with progressive aortic disease had increased serum concentrations of Macrophage Colony Stimulating Factor [14].

All these findings suggest a role for inflammation in the pathophysiology of aortic aneurysm formation in Marfan syndrome. However, it is still unclear whether these inflammatory reactions are the cause or the consequence of aortic disease. To interfere with inflammation, we studied three anti-inflammatory drugs in adult *FBN1* ^C1039G/+^ Marfan mice. Losartan is known to have AT1R-dependent anti-inflammatory effects on the vessel wall [15], and has proven effectiveness on aortic root dilatation upon long term treatment in this Marfan mouse model [7], [16]. Besides losartan, we will investigate the effectiveness of two anti-inflammatory agents that have never been applied in Marfan mice, namely the immunosuppressive corticosteroid methylprednisolone and T-cell activation blocker abatacept. Methylprednisolone preferentially binds to the ubiquitously expressed glucocorticoid receptor, a nuclear receptor, modifying inflammatory gene transcription. Abatacept is a CTLA4-Ig fusion protein that selectively binds T-cells to block CD28-CD80/86 co-stimulatory activation by MHC-II positive dendritic cells and macrophages.

In this study, we investigate the effect of these three anti-inflammatory agents on the aortic root dilatation rate, the inflammatory response in the aortic vessel wall, and Smad2 activation in adult Marfan mice.

## Methods

### Animal and study design

*FBN1* ^C1039G/+^ Marfan mice have a C57Bl6J background and are maintained as a heterozygous breeding colony in our animal facility. For breeding we used wildtype females and Marfan males to prevent death of Marfan females during pregnancy and labor. The mice included in the treatment groups were an equal mix between males and females. Polymerase chain reaction (PCR) was used to identify Marfan mice and wildtype littermates. Mice were housed in a temperature-controlled environment with 12 hour light/dark cycles and had access to food and water ad libitum. All animal protocols were approved by the Institutional Animal Welfare Committee of the Academic Medical Centre Amsterdam in the Netherlands.

Treatment was started at 8 weeks of age and was continued for 8 weeks. There was no difference in weight between Marfan and wildtype mice (males and females together and equal distribution per group; 32±19 gram versus 28±19 gram, respectively, p = 0.243). Treatment dosage in the losartan group was 0.6 g/L orally given in drinking water, which was used in previous studies [7], [16]. The two novel anti-inflammatory treatment groups received methylprednisolone 12 mg/kg or abatacept 10 mg/kg based on equal dosage in humans and previously documented dosages in mice [17]–[19]. The mice were injected three times a week by intraperitoneal (i.p.) injections of 300 µL each time. Placebo-treated Marfan mice were 1) injected i.p., three times a week with saline or 2) were not treated at all. There was no difference between the two Marfan placebo groups on aortic dilatation, medial area and elastic lamina breaks and therefore the groups were pooled. All groups contained n = 11 mice per group, except the Marfan placebo group, which consisted of n = 12 mice. At the end of the treatment period, the mice were sacrificed by an overdose of ketamine/xylazine anesthesia. Subsequently, the mice were slowly perfused with phosphate-buffered saline (PBS; 1 min) and fixative (1∶5 diluted Shandon Formal-Fixx (Thermo Scientific); 1 min), through the heart. As a reference for baseline aortic dimensions and to be able to calculate the aortic root dilatation rate, wildtype and Marfan mice were sacrificed at 8 weeks of age.

### Histology and Immunohistochemistry

Specimens of mouse hearts, containing the aortic root and part of the ascending aorta, were stored in fixative overnight at 4°C. Tissues were embedded in paraffin and then sectioned from the middle of the heart (around the mitral valves) towards the aortic arch into 7 µm sections and used for histological analyses. A standardized reference point for aortic root diameter quantification was set at the first section of the aortic root where the valve leaflets (or remnants) were not present any longer. To perform immunohistochemistry, consecutive sections were taken at this specific location.

Sections were stained with hematoxylin and eosin and were photographed (Leica Microsystem, QWin software). Image analysis software (Adobe Photoshop CS5) was used to measure the aortic wall thickness (medial area) and the aortic root perimeter (luminal circumference). The luminal aorta diameter was calculated from the perimeter. The cell nuclei were counted in two views with 200× amplification. To visualize the elastic fibers of the aortic wall, sections were stained with Lawson stain. The degree of fragmentation of the elastic fibers was examined by a pathologist (TR) blinded to the genotype and treatment group. The number of elastic lamina breaks was counted within the aortic media of each mouse. Alcian blue staining was performed to visualize acidic polysaccharide accumulation, such as glycosaminoglycans, at areas of aortic damage and quantified (corrected for medial area) with QWin software (Leica Microsystem). Nuclear Fast red was used as counterstain for nuclei.

Immunohistochemical examinations were carried out after deparaffinization and rehydration. Endogenous peroxidase activity was quenched by 20-minute incubation in 1% H_2_O_2_ and epitope retrieval was heat-induced for 10 minutes in citrate buffer pH6. Tissue sections were incubated overnight at 4°C with antibodies recognizing CD45 (clone 30 F-11, eBioscience), Mac3 (M3/84, Pharmingen) or pSmad2 (kindly provided by Peter ten Dijke) [20]. The sections were subsequently washed in tris buffered saline (TBS) and incubated with a rabbit anti-rat IgG secondary antibody (DAKO E0468) for 1 h (for CD45). The sections were incubated for 30 minutes with a horseradish peroxidase (HRP) conjugated anti-rabbit-IgG polymer (BrightVision, ImmunoLogic) for CD45 and pSmad2 and with HRP-conjugated donkey anti-rat-antibody (Jackson Lab) for Mac3. After washing, antigen detection was performed by development with diaminobenzidine tetrachloride (DAB). The sections were then mounted in Pertex and analyzed. The presence of CD45, Mac3 and pSmad2 was quantified by QWin software and expressed as positive area corrected for the total aortic wall (expressed in arbitrary units (AU)), including the intima, media and adventitia. As negative control we used normal rabbit serum, diluted similarly as the pSmad2 antiserum, which revealed no nuclear staining (data not shown). Due to the limited number of sections at the specific aortic root location, we measured one section per mouse for each staining, n≥11 per group, by an investigator blinded to the treatment.

### Statistical analysis

Statistical analysis was performed using the Kruskal–Wallis one-way analysis of variance. When the Kruskal-Wallis test leads to significant results, the two-sided Mann-Whitney U test was performed. The Spearman's rank correlation was used for the correlation between CD45 or Mac3 and aortic dilatation rate. Data are presented as median ± range. The CD45, Mac3, alcian blue and pSmad2 measurements are plotted on log scale to improve the comparison, the horizontal lines reflect the median, and the vertical lines reflect the minimum and maximum measured values. Since we compared two novel treatment groups, p<0.025 was considered statistically significant. Data analysis was performed using the SPSS statistical package (19.0 for Windows; SPSS Inc., Chicago, Illinois, USA).

## Results

### Increased inflammation in *FBN1* ^C1039G/+^ Marfan mouse aortic root

**Figure 1:**
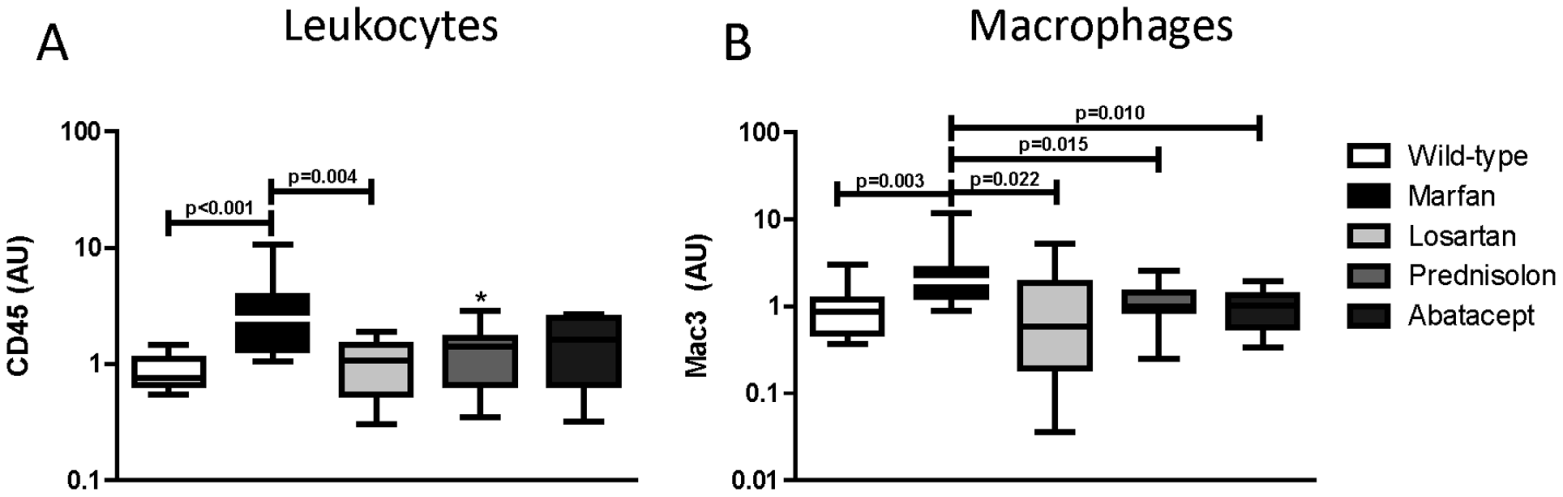
Inflammatory cells in the aortic vessel wall. Immunohistochemical staining (positive area/total aortic wall area) for leukocytes (A; CD45) and macrophages (B; Mac3) revealed that placebo-treated Marfan mice contained significantly more leukocytes and macrophages in the aortic wall as compared to wildtype mice. Losartan significantly reduced both leukocyte and macrophage influx. While methylprednisolone revealed a trend in decreased leukocytes (* p = 0.050), abatacept did not. Yet, all drugs significantly decreased the macrophage influx. Each group of mice comprises 11 mice, except Marfan placebo with n = 12, with equal male/female distribution.

To evaluate the presence of inflammation in the *FBN1* ^C1039G/+^ Marfan mouse model, we quantified the presence of leukocyte and macrophage migration into the medial and adventitial layer of the aortic wall (Figs. 1 and S1). Leukocyte migration (CD45) into the aortic wall was significantly increased in the Marfan placebo group as compared to wildtype mice (2.4±10 versus 0.8±1, p<0.001; Fig. 1A). Macrophages influx (Mac3) is considered detrimental to vascular integrity and these inflammatory cells were thus specifically analyzed. Significantly more macrophages were present in the vessel wall of the Marfan placebo mice as compared to the wildtype mice (1.9±11 versus 0.9±3, p = 0.003; Fig. 1B).

### Aortic histology upon anti-inflammatory treatment

Leukocyte migration (CD45) into the aortic wall was significantly reduced by losartan (1.1±2, p = 0.004). Methylprednisolone (1.4±3, p = 0.050) and abatacept (1.6±2, p = 0.149), did not reduce leukocyte infiltration significantly, when compared to Marfan placebo mice (Fig. 1A), although methylprednisolone showed a trend. However, macrophage influx was significantly reduced by losartan (0.6±5, p = 0.022), methylprednisolone (1.0±2, p = 0.015) as well as by abatacept (1.0±2, p = 0.010) (Fig. 1B). Thus, the 2 novel anti-inflammatory treatment strategies predominantly reduce macrophage influx into the vessel wall.

**Figure 2:**
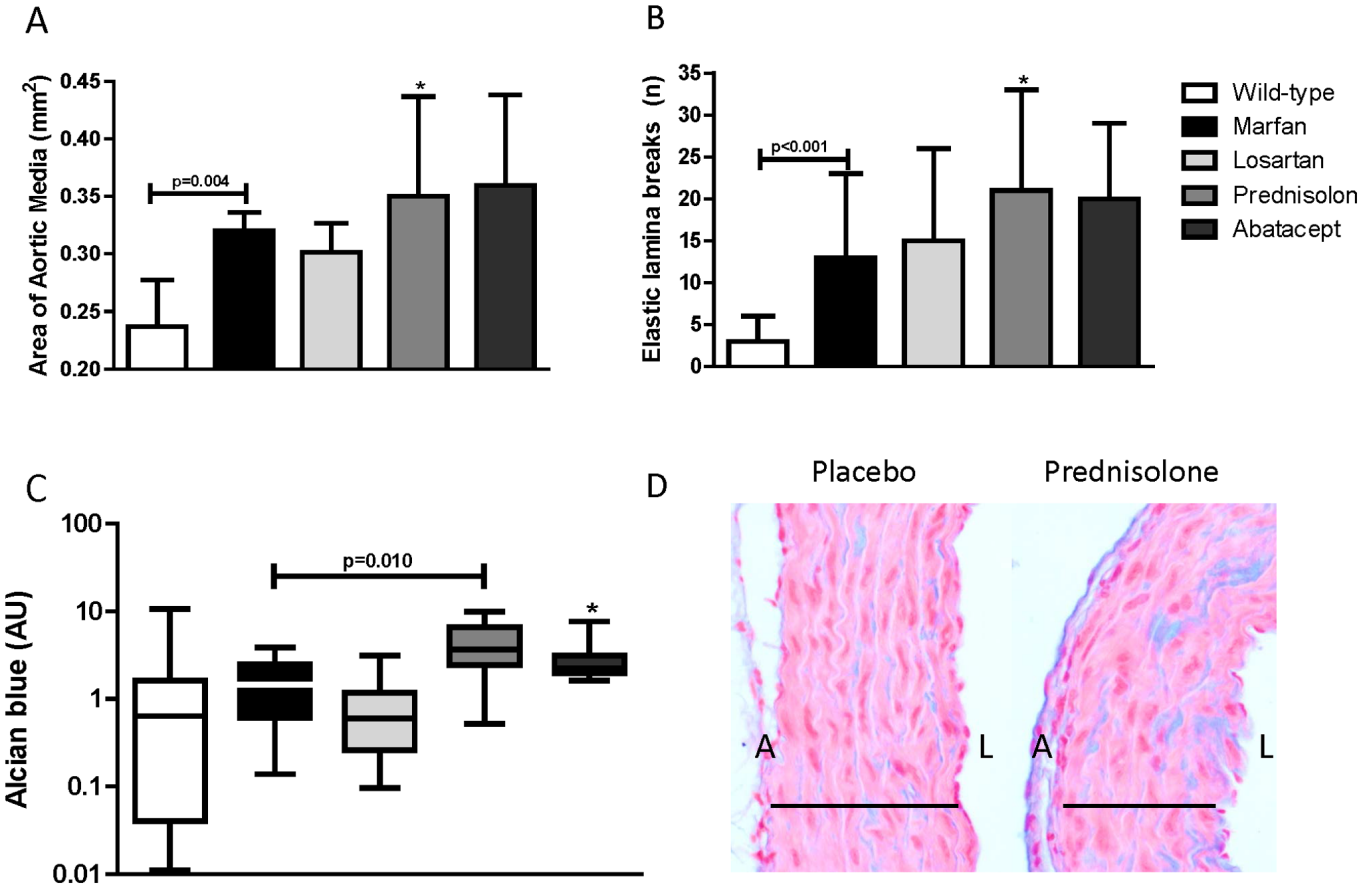
Aortic wall thickness, elastin breaks and GAG accumulation. A) The area of the aortic media of placebo-treated Marfan mice was significantly thickened compared to wall thickness in wildtype mice. Methylprednisolone showed a trend towards enhanced thickening of the aortic media in Marfan mice (* p = 0.066). B) There were significantly more elastic lamina breaks in the aortic wall of Marfan mice compared to wildtype mice. Methylprednisolone revealed a trend towards enhanced elastic lamina breaks in the aortic media in Marfan mice (* p = 0.076). C) There was enhanced alcian blue positive area in the aortic media of methylprednisolone-treated mice, as compared to Marfan placebo mice, as a marker for medial necrosis. Abatacept showed a trend towards increased GAG accumulation as visualized by alcian blue (* p = 0.066). D) Alcian blue staining (blue) is present in the media (black line) in placebo-treated Marfan mice, yet it is more pronounced in the Methylprednisolone-treated aortic root. Pink stain = cytoplasm, red dots = nuclei, A = adventitia, L = lumen.

As a measure of changed morphology of the vessel wall, we determined the thickness of the smooth muscle cell containing medial layer of the aortic root (Fig. 2A). The area of the aortic media was significantly increased in Marfan mice, compared to wildtype mice (0.32±0.1 versus 0.24±0.1 mm^2^, p = 0.004), which was not changed by losartan (0.30±0.1 mm^2^, p = 0.767). Abatacept did not show a difference (0.36±0.2 mm^2^, p = 0.148), while methylprednisolone showed a trend towards an increased thickness of the medial layer (0.35±0.4 mm^2^, p = 0.066). The number of smooth muscle cells (cell nuclei) were similar between untreated Marfan mice (227±19 cell nuclei) and methylprednisolone- (220±28 cell nuclei, p = 0.674) or abatacept-treated Marfan mice (231±28 cell nuclei, p = 0.786).

The degree of elastic lamina breaks in the medial layer is a measure of vascular damage and was compared between treatment groups. Placebo-treated Marfan mice showed a significant increase in elastic lamina breaks (Marfan: 12±20 versus wildtype: 3±9, p<0.001) (Fig. 2B). None of the treatment groups preserved the vascular integrity by decreasing the number of elastic lamina breaks within the medial layer. However, methylprednisolone showed a trend towards increased number of elastic lamina breaks (25±31, p = 0.076).

In Marfan patients, it is known that alcian blue staining detects areas of cystic medial necrosis.^21^ At sites of smooth muscle cell death and elastic lamina breaks, acidic polysaccharides such as glycosaminoglycans (GAG) accumulate. Therefore, alcian blue staining is performed to visualize the medial necrosis in the various Marfan treatment groups (Fig. 2C,D). Interestingly, the methylprednisolone group showed a significant increase in alcian blue staining as compared to the Marfan placebo-treated mice (p = 0.010), and abatacept revealed a trend in increased GAG accumulation (p = 0.066), suggesting that these anti-inflammatory treatment strategies are potentially harmful. In conclusion, all anti-inflammatory treatment groups, including losartan, revealed decreased macrophages within the aortic wall, but none of these drugs improved aorta morphology in this short time frame. Methylprednisolone-treated mice seemed to have even more aortic damage.

### Losartan inhibits the aortic dilatation rate, which is not affected by the other drugs

To study whether all three anti-inflammatory drugs used in this study have an effect on aortic root dilatation in Marfan syndrome, we measured the aortic root diameters in tissue sections. Losartan showed a protective effect on aortic root dilatation when treatment started at 6 weeks of age and persisted during 6.5 months [7], [16]. We started treatment in adult mice at 8 weeks of age. The Marfan mice then already showed a significant increase in aortic root diameter when compared to wildtype littermates (0.62 mm±0.09 versus 0.55 mm±0.10, p = 0.007). After a treatment period of only 8 weeks, the aortic root diameter was dilated more pronounced in placebo-treated Marfan mice compared to the diameter of wildtype mice (1.15 mm±0.21 versus 0.98 mm±0.27, respectively, p<0.001). Losartan could significantly attenuate aortic root diameter enlargement in this short time frame in Marfan mice (1.09 mm±0.23, p = 0.023). However, methylprednisolone (1.15 mm±0.37, p = 0.898) and abatacept (1.21 mm±0.46, p = 0.847) did not inhibit aortic root dilatation.

**Figure 3:**
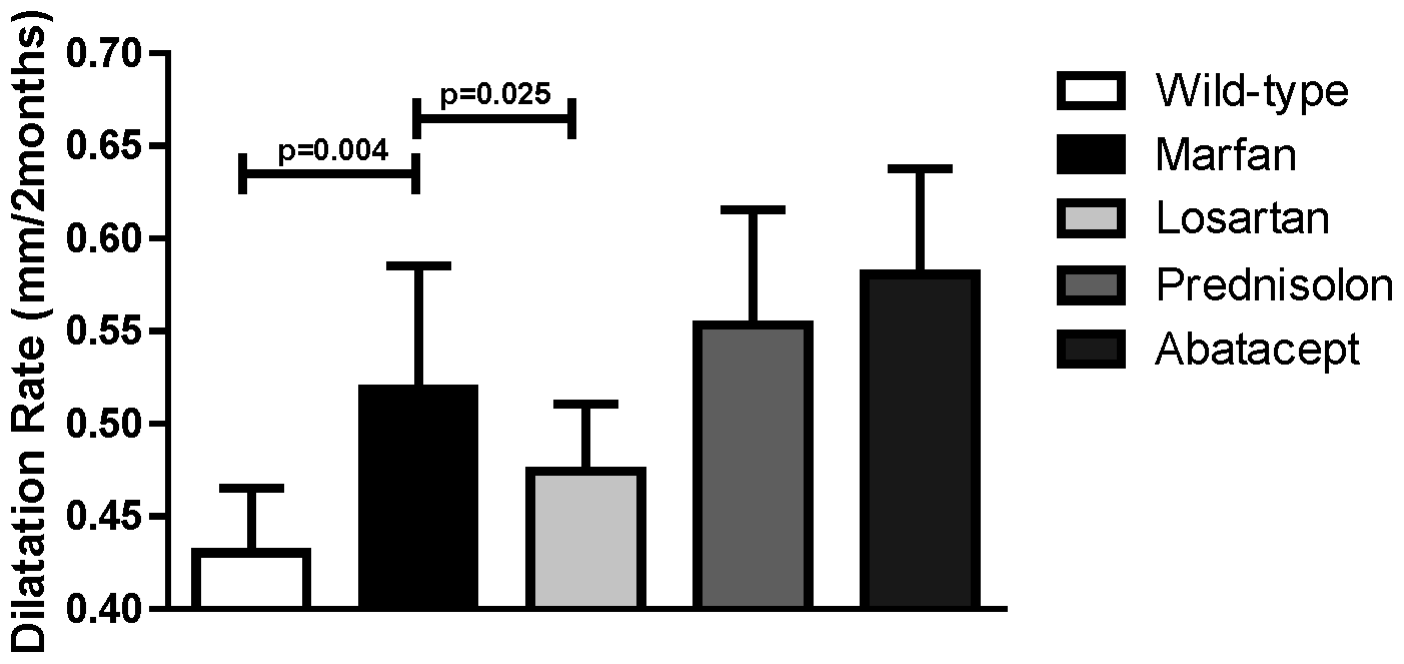
Aortic dilatation in Marfan mice reduced by losartan. The aortic root dilatation rate was determined. Placebo-treated Marfan mice had a significantly higher dilatation rate compared to wildtype mice. Losartan attenuated the aortic root dilatation rate in Marfan mice significantly, whereas the other treatment strategies did not change the aortic root dilatation rate compared to placebo-treated Marfan mice.

We calculated the aortic root dilatation rate by using the aortic root diameters of wildtype and Marfan mice that were sacrificed at the age of 8 weeks old (initiation of treatment) and 16 weeks old (termination of treatment). Placebo-treated Marfan mice demonstrated a significantly increased aortic root dilatation rate, when compared to wildtype mice (+0.52±0.24 mm/2 months versus +0.43±0.25 mm/2 months, p = 0.004; Fig 3). Losartan was again the only drug that inhibited the aortic root dilatation rate significantly (+0.47±0.25, p = 0.025). Methylprednisolone and abatacept did not show any significant change in the aortic root dilatation rate when compared to placebo-treated Marfan mice (+0.55±0.34, p = 0.848 and +0.58±0.43, p = 0.876, respectively).

For the correlation between inflammation and aortic root diameter/aortic root dilatation rate we included each individual mouse of this experiment. As expected from earlier observations in human Marfan patients and the mgR Marfan mice, the number of leukocytes in the vessel wall (CD45) correlates with aortic root diameter (r = 0.563, p<0.001), and with aortic root dilatation rate (r = 0.405, p = 0.003). The number of infiltrated macrophages (Mac3) correlates with aortic root diameter (r = 0.304, p = 0.012), but surprisingly not with aortic root dilatation rate (r = 0.185, p = 0.177).

### Aortic Smad2 signaling

**Figure 4:**
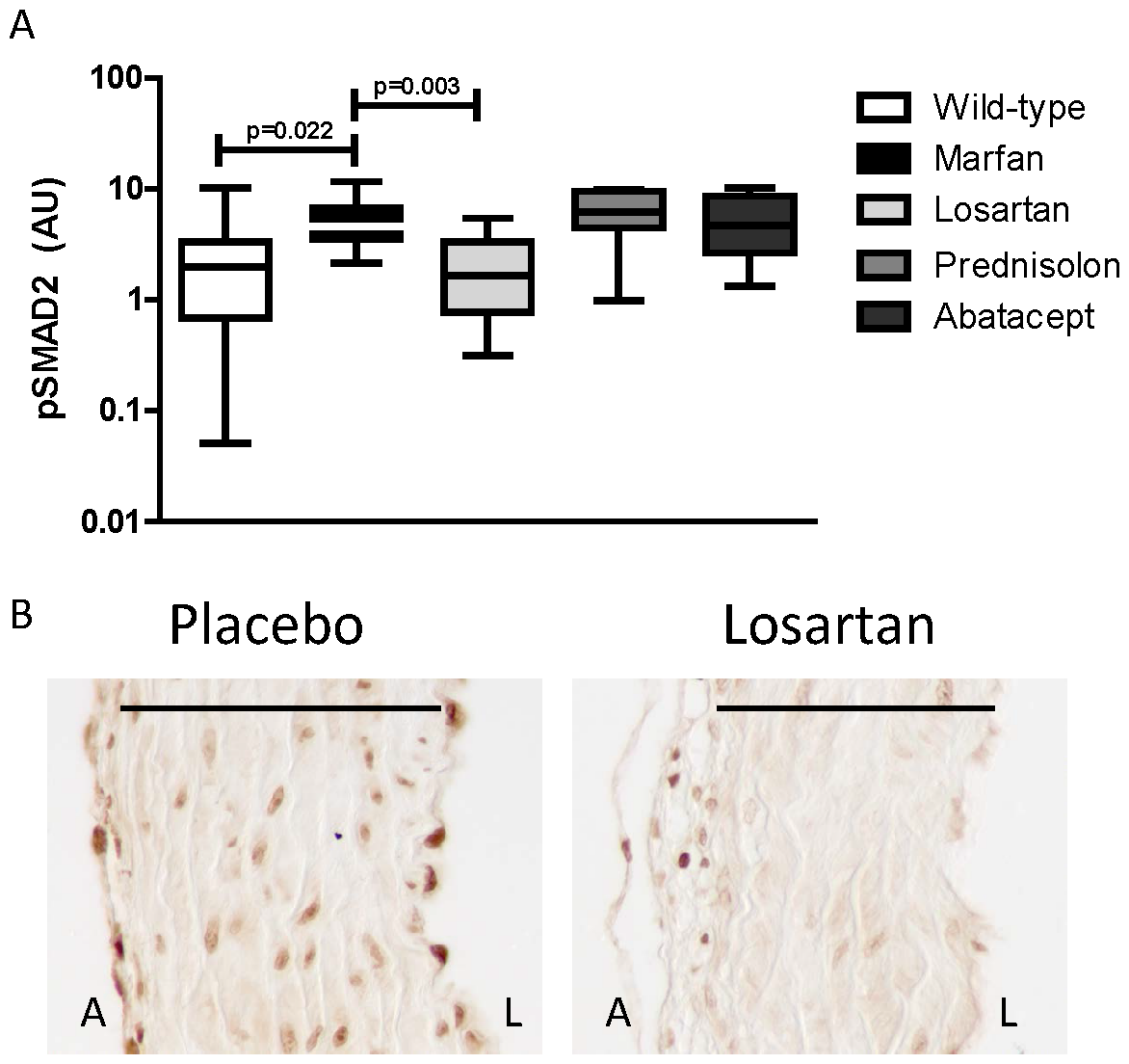
Aortic SMAD2 signaling. A) Phosphorylation of Smad2 (pSmad2) and localization in the nucleus of vascular cells in the aortic wall (positive area/total aortic wall area) is expressed in arbitrary units (AU). pSmad2 was significantly reduced by losartan treatment, as compared to placebo-treated Marfan mice. The other anti-inflammatory drugs did not affect the number of pSmad2-positive nuclei. B) An example of pSmad2 staining in placebo-treated Marfan mice and reduced pSmad2 in losartan-treated Marfan mice. A = adventitia, L = lumen, line indicates media.

**Figure 5:**
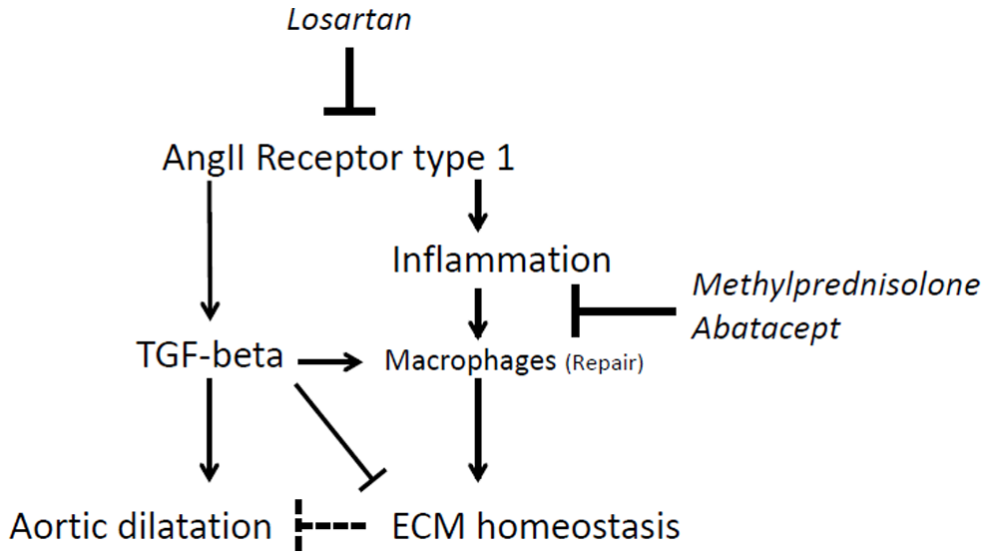
Proposed mechanism. Losartan is currently the only drug that effectively inhibits aortic root dilatation in mice and men, and specifically targets the angiotensin-II receptor type 1. Losartan clearly decreases TGF-β/pSmad2 signaling, decreases total leukocyte and macrophage influx into the vessel wall, and diminishes aortic root dilatation. TGF-β is known to polarize macrophages into a repair phenotype and at the same time induces collagen synthesis and matrix metalloproteinase activity to degrade extracellular matrix proteins (ECM). Methylprednisolone and abatacept decreased macrophage influx significantly, which resulted in increased GAG accumulation in the aortic vessel wall, thus disturbing ECM homeostasis, which may be potentially harmful.

AT1R and TGF-β signaling are considered detrimental in Marfan syndrome; therefore we also investigated activation of its downstream transcription factor Smad2 in the aortic root. We measured phosphorylated Smad2 (pSmad2) in the nucleus of aortic endothelial cells (intima), smooth muscle cells (media) and fibroblasts (adventitia) and inflammatory cells locally present. In placebo-treated Marfan mice, nuclear pSmad2 was increased compared to wildtype littermates (4.0±11 versus 2.8±10, p = 0.022, Fig. 4A). Methylprednisolone or abatacept did not show a change in pSmad2 compared to placebo-treated Marfan mice (6.2±9, p = 0.511 and 4.7±9, p = 0.793, respectively). Significantly, losartan decreased nuclear pSmad2 staining (1.6±5, p = 0.003), which is almost absent in the smooth muscle cells (Fig. 4B). In conclusion, where all three anti-inflammatory treatments responded equally in decreasing the macrophage influx into the aortic wall, a decrease in total leukocytes or pSmad2 was only observed in the losartan-treated mice. We hypothesize that a reduced macrophage influx alone interferes with extracellular matrix homeostasis, while additional suppression of leukocyte influx and pSmad2 signaling reduces aortic dilatation (Fig. 5).

## Discussion

In the present study we showed increased vascular inflammation in the aortic root of adult Marfan mice, which was significantly reduced by short term losartan treatment, accompanied by decreased nuclear pSmad2 in the vessel wall and prevention of aortic root dilatation.

We demonstrate that the increased inflammatory profile of the human Marfan aorta is also observed in the aortic vessel wall of adult *FBN1* ^C1039G/+^ Marfan mice. Therefore, we chose to intervene with the established general anti-inflammatory drug methylprednisolone which activates the glucocorticoid receptor that is protective in vascular disease, as summarized in a recent review [21]. When treating Marfan mice with methylprednisolone, a significant decrease in macrophage influx was demonstrated. However, an increase in GAG accumulation was observed, while the aortic dilatation rate remained the same. This indicates that glucocorticoids should not become the drug of choice to prevent aortic dilatation in Marfan syndrome, especially when taking into consideration that these drugs have severe side effects in chronic use.

We previously revealed that MHC-II genes *HLA-DRB1* and *HLA-DRB5* correlate in Marfan patients with an increased aortic root dilatation rate [14]. Therefore, we choose to treat Marfan mice with abatacept, which blocks T-cell activation by MHC-II positive antigen presenting cells. Abatacept has been shown to effectively inhibit atherosclerosis in mice [22] and to reduce renin-angiotensin-aldosterone (RAAS)-induced hypertension [23]. In Marfan mice, abatacept treatment resulted in a decreased macrophage influx into the aorta, yet abatacept did not protect from aortic dilatation.

An underestimated aspect of vascular inflammation is the variety in inflammatory responses. Vascular inflammation either promotes or repairs damage [24], [25]. Here, we observed an increased influx of inflammatory cells in Marfan placebo mice, and a clear correlation between leukocyte presence in the vessel wall and aortic dilatation rate. Yet, a correlation between macrophages and aortic dilatation rate was not significant, while methylprednisolone and abatacept predominantly reduced macrophage influx. Even though we did not further characterize the leukocyte populations, it seems that leukocytes, other than macrophages, may be detrimental in aortic dilatation, while the macrophages may promote vascular repair in Marfan syndrome. In immunology, TGF-β (abundantly present in Marfan [26]) is mostly known as an anti-inflammatory factor, promoting resolution of inflammation by skewing macrophages towards a protective “repair” phenotype [27].

The increased accumulation of GAG in the aortic media of methylprednisolone-treated mice, suggests that there is increased vascular damage upon use of this immunosuppressive drug, which may be harmful upon long term treatment. In line with these data, Lindeman *et al.* presented a case study in which a patient with an abdominal aortic aneurysm (AAA) had a sudden increase in aortic dilatation rate (from 3.4 cm to 7.0 cm in 27 months) upon immunosuppressive therapy (combination therapy containing glucocorticoids) after kidney transplantation [28]. In addition, in 18 patients with abdominal or thoracic aneurysms, the aneurysm dilatation rate was increased from 0.46 cm/year before transplantation to 1.0 cm/year after transplant operation and the start of immunosuppressive drugs [29]. Similarly, in the Blotchy mouse aneurysm model, aortic rupture occurred upon glucocorticoid treatment [30]. So, based on these and our data, a similar phenomenon may occur in Marfan patients with existing aorta dilatation, when using glucocorticoids. In general, the anti-inflammatory drugs did not contribute to the improvement of aorta pathology in Marfan mice, suggesting that the macrophage influx is rather a consequence of aortic damage than the cause of aortic dilatation in Marfan syndrome. However, a beneficial effect of the anti-inflammatory drugs after longer treatment or in older Marfan mice with more severe aortic inflammation cannot be excluded.

In this 8-week treatment period in adult Marfan mice, losartan consistently reduced vascular inflammation, nuclear pSmad2 and most importantly aortic root dilatation, despite lack of improvement in medial thickness or elastin breaks. Our treatment strategy could therefore be considered as a rapid screening approach for novel drugs in Marfan syndrome. Losartan is the first treatment targeting the underlying aortic pathophysiology in Marfan syndrome and is effective in reducing aortic dilatation rate in patients and mice with Marfan syndrome [7], [9]. Losartan is an AT1R-blocker, which counteracts the effect of angiotensin II-mediated detrimental signaling cascades; including TGF-β production, pSmad2 signaling, increasing blood pressure, reactive oxygen species generation, and induction of a pro-inflammatory response [31]–[33]. Thus increased leukocytes (other than macrophages) and TGF-β/pSmad2 by angiotensin II-induced signaling seems to be the underlying devastating pathway of Marfan syndrome [34]. Recently, a study has demonstrated epigenetic changes in the Smad2 promoter in vascular smooth muscle cells derived from human thoracic aneurysm tissue [35]. This study highlights the important role of Smad2 and TGF-β in thoracic aortic aneurysms. In addition, mutations in the TGF-β receptor genes (TGFBR1 and TGFBR2) result in Marfan-like syndromes with aortic aneurysms and dissections as well, named ‘Loeys-Dietz Syndrome’ [36].

Besides losartan therapy, doxycycline, an antibiotic with anti-inflammatory and matrix metalloproteinases (MMP) inhibition capacities [37], reduced aortic root dilatation rate in two different mouse models of Marfan syndrome (*FBN1* ^C1039G/+^ and *FBN1* ^mgR/mgR^) [38]–[40]. It has been suggested that doxycycline reduces aortic root dilatation rate through the TGF-β and pSmad2 pathway [38]–[41]. TGF-β stimulates the expression of MMP in vascular cells. Furthermore, MMP can activate TGF-β through proteolytic degradation of the latent TGF-β complex [42]. In conclusion, doxycycline may reduce aortic dilatation rate in Marfan mice by inhibiting TGF-β-induced MMP production and by inhibiting MMP-induced release of TGF-β, rather than by reducing inflammation. However, in the only trial in patients with aneurysms, doxycycline presented an unexpected increase in aortic diameter of 0.8 mm after 18 months, when compared to the placebo AAA patients [43].

In conclusion, the use of anti-inflammatory drugs methylprednisolone and abatacept did not protect against progressive aortic root dilatation in Marfan mice. So far, losartan is the only drug that can prevent aortic dilatation in adult Marfan mice and patients. Inhibition of macrophage influx did not reduce the aortic diameter and aortic root dilatation rate. Thus, macrophages do not seem to play a major role in promoting aortic pathology. Hence, inhibition of inflammation may be potentially harmful in Marfan patients. When long-term immunosuppressive therapy is needed in Marfan patients, the aorta should be carefully monitored for excessive dilatation.

## Supporting Information

Figure S1 **Leukocyte and macrophage presence in the aortic root.** Left panel: Leukocytes (CD45) were hardly detectable in wildtype (WT) mice, whereas leukocytes were present (dark brown) in the Marfan (MFS) aorta, mostly in the intima at the lumen side (L) or in the adventitia (A). Right panel: Macrophages (MAC3) were barely located in wildtype mice (dark brown), but were observed in the Marfan mice, occasionally within the aortic media (black line). blue dots =  nuclei.
(TIF)

## References

[1] Franken R, Hartog AW, Singh M, Pals G, Zwinderman AH, et al (2013) Marfan syndrome - Progress report. Progress in Pediatric Cardiology 34: 9–14 10.1016/j.ppedcard.2012.05.003

[2] de Witte P, Aalberts JJ, Radonic T, Timmermans J, Scholte AJ, et al (2011) Intrinsic biventricular dysfunction in Marfan syndrome. Heart 97: 2063–2068 10.1136/heartjnl-2011-300169 21990385

[3] Engelfriet PM, Boersma E, Tijssen JG, Bouma BJ, Mulder BJ (2006) Beyond the root: dilatation of the distal aorta in Marfan's syndrome. Heart 92: 1238–1243 10.1136/hrt.2005.081638 16488927 PMC1861183

[4] Hartog AW, Franken R, Zwinderman AH, Groenink M, Mulder BJ (2012) Current and future pharmacological treatment strategies with regard to aortic disease in Marfan syndrome. Expert Opin Pharmacother 13: 647–662 10.1517/14656566.2012.665446 22397493

[5] Meijboom LJ, Groenink M, van der Wall EE, Romkes H, Stoker J, et al (2000) Aortic root asymmetry in marfan patients; evaluation by magnetic resonance imaging and comparison with standard echocardiography. Int J Card Imaging 16: 161–168. 11144769 10.1023/a:1006429603062

[6] Meijboom LJ, Timmermans J, Zwinderman AH, Engelfriet PM, Mulder BJ (2005) Aortic root growth in men and women with the Marfan's syndrome. Am J Cardiol 96: 1441–1444 10.1016/j.amjcard.2005.06.094 16275195

[7] Habashi JP, Judge DP, Holm TM, Cohn RD, Loeys BL, et al (2006) Losartan, an AT1 antagonist, prevents aortic aneurysm in a mouse model of Marfan syndrome. Science 312: 117–121 10.1126/science.1124287 16601194 PMC1482474

[8] Kim KL, Yang JH, Song SH, Kim JY, Jang SY, et al (2013) Positive correlation between the dysregulation of transforming growth factor-beta1 and aneurysmal pathological changes in patients with Marfan syndrome. Circ J 77 952–958: doi.org/10.1253/circj.CJ–12-0874 10.1253/circj.cj-12-0874 23291965

[9] Groenink M, den Hartog AW, Franken R, Radonic T, de Waard V, et al (2013) Losartan reduces aortic dilatation rate in adults with Marfan syndrome: a randomized controlled trial. Eur Heart J 34: 3491–3500 10.1093/eurheartj/eht334 23999449

[10] Pereira L, Lee SY, Gayraud B, Andrikopoulos K, Shapiro SD, et al (1999) Pathogenetic sequence for aneurysm revealed in mice underexpressing fibrillin-1. Proc Natl Acad Sci U S A 96: 3819–3823 10.1073/pnas.96.7.3819 10097121 PMC22378

[11] Guo G, Booms P, Halushka M, Dietz HC, Ney A, et al (2006) Induction of macrophage chemotaxis by aortic extracts of the mgR Marfan mouse model and a GxxPG-containing fibrillin-1 fragment. Circulation 114: 1855–1862 10.1161/CIRCULATIONAHA.105.601674 17030689

[12] Guo G, Gehle P, Doelken S, Martin-Ventura JL, von Kodolitsch Y, et al (2011) Induction of macrophage chemotaxis by aortic extracts from patients with Marfan syndrome is related to elastin binding protein. PLoS One 6: e20138 10.1371/journal.pone.0020138 21647416 PMC3103536

[13] He R, Guo DC, Estrera AL, Safi HJ, Huynh TT, et al (2006) Characterization of the inflammatory and apoptotic cells in the aortas of patients with ascending thoracic aortic aneurysms and dissections. J Thorac Cardiovasc Surg131: 671–678 10.1016/j.jtcvs.2005.09.018 16515922

[14] Radonic T, de Witte P, Groenink M, de Waard V, Lutter R, et al (2012) Inflammation aggravates disease severity in Marfan syndrome patients. PLoS One 7: e32963 10.1371/journal.pone.0032963 22479353 PMC3316543

[15] Dai Q, Xu M, Yao M, Sun B (2007) Angiotensin AT1 receptor antagonists exert anti-inflammatory effects in spontaneously hypertensive rats. Br J Pharmacol 152: 1042–1048 10.1038/sj.bjp.0707454 17922026 PMC2095108

[16] McLoughlin D, McGuinness J, Byrne J, Terzo E, Huuskonen V, et al (2011) Pravastatin reduces Marfan aortic dilation. Circulation 124: S168–S173 10.1161/CIRCULATIONAHA.110.012187 21911808

[17] Liu Z, Bethunaickan R, Huang W, Ramanujam M, Madaio MP, et al (2011) IFN-α confers resistance of systemic lupus erythematosus nephritis to therapy in NZB/W F1 mice. J Immunol 187: 1506–1513 10.4049/jimmunol.1004142 21705616 PMC3140572

[18] Christensen AD, Skov S, Haase C (2013) Local and systemic effects of co-stimulatory blockade using cytotoxic T lymphocyte antigen-4-immunoglobulin in dinitrofluorobenzene- and oxazolone-induced contact hypersensitivity in mice. Clin Exp Immunol 171: 220–230 10.1111/cei.12005 23286949 PMC3573293

[19] Bassett D, Hirata F, Gao X, Kannan R, Kerr J, et al (2010) Reversal of methylprednisolone effects in allergen-exposed female BALB/c mice. J Toxicol Environ Health A. 2010 73(11): 711–24 10.1080/15287391003614018 20391114

[20] Persson U, Izumi H, Souchelnytskyi S, Itoh S, Grimsby S, et al (1998) The L45 loop in type I receptors for TGF-beta family members is a critical determinant in specifying Smad isoform activation. FEBS Lett 434: 83–87 10.1016/S0014-5793(98)00954-5 9738456

[21] Kurakula K, Hamers AA, de Waard V, de Vries CJ (2013) Nuclear Receptors in atherosclerosis: a superfamily with many ‘Goodfellas’. Mol Cell Endocrinol 368: 71–84 10.1016/j.mce.2012.05.014 22664910

[22] Ewing MM, Karper JC, Abdul S, de Jong RC, Peters HA, et al (2013) T-cell co-stimulation by CD28-CD80/86 and its negative regulator CTLA-4 strongly influence accelerated atherosclerosis development. Int J Cardiol 168: 1965–1974 10.1016/j.ijcard.2012.12.085 23351788

[23] Vinh A, Chen W, Blinder Y, Weiss D, Taylor WR, et al (2010) Inhibition and genetic ablation of the B7/CD28 T-cell costimulation axis prevents experimental hypertension. Circulation 122: 2529–2537 10.1161/CIRCULATIONAHA.109.930446 21126972 PMC3064430

[24] Nataatmadja M, West M, West J, Summers K, Walker P, et al (2003) Abnormal extracellular matrix protein transport associated with increased apoptosis of vascular smooth muscle cells in marfan syndrome and bicuspid aortic valve thoracic aortic aneurysm. Circulation 108: II329–334 10.1161/01.cir.0000087660.82721.15 12970255

[25] Malhotra N, Kang J (2013) SMAD regulatory networks construct a balanced immune system. Immunology 139: 1–10 10.1111/imm.12076 23347175 PMC3634534

[26] Franken R, den Hartog AW, de Waard V, Engele L, Radonic T, et al (2013) Circulating transforming growth factor-beta as a prognostic biomarker in Marfan syndrome. Int J Cardiol 168: 2441–2446 10.1016/j.ijcard.2013.03.033 23582687

[27] Mantovani A, Biswas SK, Galdiero MR, Sica A, Locati M (2013) Macrophage plasticity and polarization in tissue repair and remodelling. J Pathol 229: 176–185 10.1002/path.4133 23096265

[28] Lindeman JH, Rabelink TJ, van Bockel JH (2011) Immunosuppression and the abdominal aortic aneurysm: Doctor Jekyll or Mister Hyde? Circulation 124: e463–e465 10.1161/CIRCULATIONAHA.110.008573 22042930

[29] Englesbe MJ, Wu AH, Clowes AW, Zierler RE (2003) The prevalence and natural history of aortic aneurysms in heart and abdominal organ transplant patients. J Vasc Surg 37: 27–31 10.1067/mva.2003.57 12514574

[30] Reilly JM, Savage EB, Brophy CM, Tilson MD (1990) Hydrocortisone rapidly induces aortic rupture in a genetically susceptible mouse. Arch Surg 125: 707–709 10.1001/archsurg.1990.01410180025004 2346371

[31] Chen X, Lu H, Rateri DL, Cassis LA, Daugherty A (2013) Conundrum of angiotensin II and TGF-beta interactions in aortic aneurysms. Curr Opin Pharmacol 13: 180–185 10.1016/j.coph.2013.01.002 23395156 PMC3691307

[32] Carvajal G, Rodriguez-Vita J, Rodrigues-Diez R, Sanchez-Lopez E, Ruperez M, et al (2008) Angiotensin II activates the Smad pathway during epithelial mesenchymal transdifferentiation. Kidney Int 74: 585–595 10.1038/ki.2008.213 18509316

[33] Rodriguez-Vita J, Sanchez-Lopez E, Esteban V, Ruperez M, Egido J, et al (2005) Angiotensin II activates the Smad pathway in vascular smooth muscle cells by a transforming growth factor-beta-independent mechanism. Circulation 111: 2509–2517 10.1161/01.CIR.0000165133.84978.E2 15883213

[34] Franken R, Radonic T, den Hartog AW, Groenink M, Pals G, et al.. (2014) The revised role of TGF-β in aortic aneurysms in Marfan syndrome. Neth Heart J. 10.1007/s12471-014-0622-0 PMC4315797 25342281

[35] Gomez D, Kessler K, Michel JB, Vranckx R (2013) Modifications of chromatin dynamics control Smad2 pathway activation in aneurysmal smooth muscle cells. Circ Res 113: 881–890 10.1161/CIRCRESAHA.113.301989 23825360

[36] Loeys BL, Chen J, Neptune ER, Judge DP, Podowski M, et al (2005) A syndrome of altered cardiovascular, craniofacial, neurocognitive and skeletal development caused by mutations in TGFBR1 or TGFBR2. Nat Genet 37: 275–281 10.1038/ng1511 15731757

[37] Lindeman JH, Abdul-Hussien H, van Bockel JH, Wolterbeek R, Kleemann R (2009) Clinical trial of doxycycline for matrix metalloproteinase-9 inhibition in patients with an abdominal aneurysm: doxycycline selectively depletes aortic wall neutrophils and cytotoxic T cells. Circulation 119: 2209–2216 10.1161/CIRCULATIONAHA.108.806505 19364980

[38] Chung AW, Yang HH, Radomski MW, van Breemen C (2008) Long-term doxycycline is more effective than atenolol to prevent thoracic aortic aneurysm in marfan syndrome through the inhibition of matrix metalloproteinase-2 and -9. Circ Res 102: e73–e85 10.1161/CIRCRESAHA.108.174367 18388324

[39] Xiong W, Knispel RA, Dietz HC, Ramirez F, Baxter BT (2008) Doxycycline delays aneurysm rupture in a mouse model of Marfan syndrome. J Vasc Surg 47: 166–172 10.1016/j.jvs.2007.09.016 18178469 PMC4148046

[40] Yang HH, Kim JM, Chum E, van Breemen C, Chung AW (2010) Effectiveness of combination of losartan potassium and doxycycline versus single-drug treatments in the secondary prevention of thoracic aortic aneurysm in Marfan syndrome. J Thorac Cardiovasc Surg 140: 305–312 10.1016/j.jtcvs.2009.10.039 20189193

[41] Kim HS, Luo L, Pflugfelder SC, Li DQ (2005) Doxycycline inhibits TGF-beta1-induced MMP-9 via Smad and MAPK pathways in human corneal epithelial cells. Invest Ophthalmol Vis Sci 46: 840–848 10.1167/iovs.04-0929 15728539

[42] Yu Q, Stamenkovic I (2000) Cell surface-localized matrix metalloproteinase-9 proteolytically activates TGF-beta and promotes tumor invasion and angiogenesis. Genes Dev 14: 163–176 10.1101/gad.14.2.163 10652271 PMC316345

[43] Meijer CA, Stijnen T, Wasser MN, Hamming JF, van Bockel JH, et al (2013) Doxycycline for stabilization of abdominal aortic aneurysms: a randomized trial. Ann Intern Med 159: 815–823 10.7326/0003-4819-159-12-201312170-00007 24490266

