# Supplementary figures and images for: No Beneficial Effect of General and Specific Anti-Inflammatory Therapies on Aortic Dilatation in Marfan Mice
Source: PLoS One. 2014 Sep 19;9(9):e107221. doi: 10.1371/journal.pone.0107221 (PMC4169510; doi:10.1371/journal.pone.0107221)

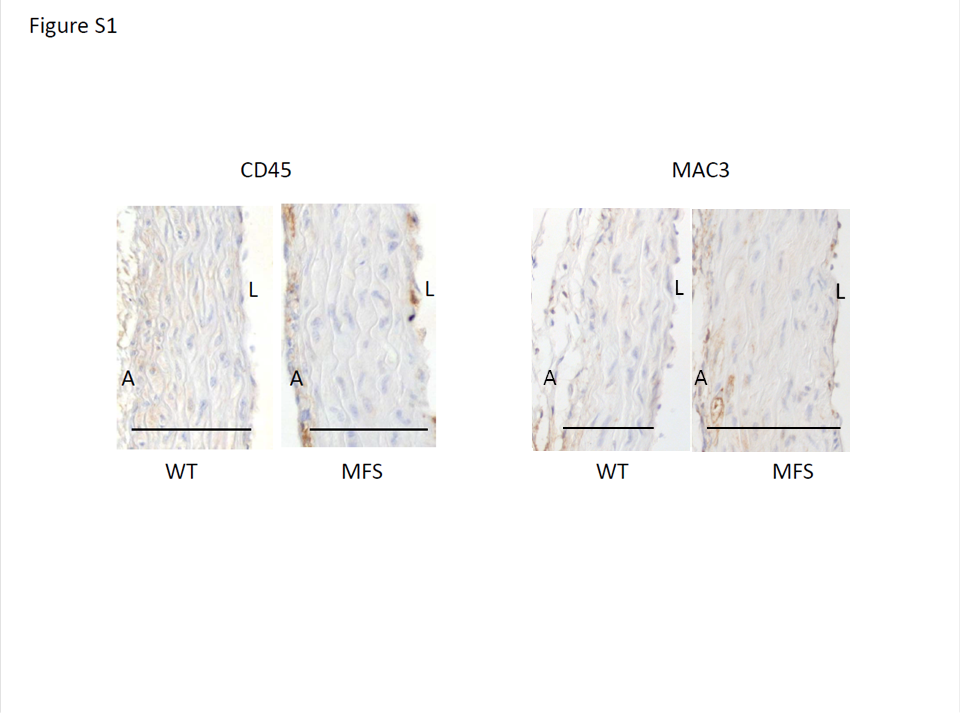

Supplement: Figure S1 — Leukocyte and macrophage presence in the aortic root. Left panel: Leukocytes (CD45) were hardly detectable in wildtype (WT) mice, whereas leukocytes were present (dark brown) in the Marfan (MFS) aorta, mostly in the intima at the lumen side (L) or in the adventitia (A). Right panel: Macrophages (MAC3) were barely located in wildtype mice (dark brown), but were observed in the Marfan mice, occasionally within the aortic media (black line). blue dots = nuclei. (TIF) [file pone.0107221.s001.tif]
